# Pharmaceutical Standardization and Physicochemical Characterization of Traditional Ayurvedic Marine Drug: Incinerated Conch Shell (*Shankha Bhasma*)

**DOI:** 10.3390/md16110450

**Published:** 2018-11-15

**Authors:** Sandeep Chavan, Sonali Tayade, Vidya Gupta, Vineeta Deshmukh, Sadanand Sardeshmukh

**Affiliations:** 1 Drug Standardization Laboratory, Bharatiya Sanskriti Darshan Trust’s Integrated Cancer Treatment and Research Centre, Wagholi, Pune 412207, India; deep.veda2018@gmail.com (S.C.); drsandeep.atharva@gmail.com (S.T.); bsdtdadar@gmail.com (V.D.); 2 Bhasma Section, Atharva Nature Healthcare Pvt. Ltd., Wagholi, Pune 412207, India; 3 CSIR-National Chemical Laboratory, Pune 411008, India; vs.gupta@ncl.res.in

**Keywords:** conch, incineration, *Shankha Bhasma*, pharmaceutical standardization, physico-chemical properties, calcium carbonate, calcite, aragonite, calcium group (*Sudha varga*) drugs

## Abstract

Natural resources such as plants, animals and minerals have always been used by mankind to develop drugs and marine world is no exception. Marine by-products like conches, pearls, mother of pearl shells, corals and so forth have been used by traditional *Ayurvedic* practitioners for centuries. The unique methods of these preparations are scientifically designed to eliminate unwanted impurities and convert them into bioavailable form. In this study, Conch (*Xanchus pyrum*) was used as a marine resource of calcium carbonate and was converted pharmaceutically from its aragonite form to calcite. All the steps of preparations and changes in the properties therein were documented and validated. Further, traditional as well as modern analytical tools were used to study its physical and chemical characters to develop a monograph. The physical characterization included particle size, X-ray diffraction (XRD), Scanning Electron Microscopy (SEM), Thermogravimetric Analysis (TGA) and Fourier Transform Infra-red (FTIR). Metal composition and heavy metal limits were determined using Inductively Coupled Plasma Optical Emission Spectrometry (ICPOES). This study revealed the rearrangement of aragonite crystals into calcite form by grinding, trituration with aloe vera juice and incineration under controlled conditions. Moreover, the finished product was found to be devoid of organic matrix that is nacre. This study creates a foundation for the development of a master formula for commonly used *Shankha Bhasma* in Ayurvedic medicines.

## 1. Introduction

Conch is a common name that is applied to a number of medium to large-sized shells of large snails (*Turbinella pyrum*) from the family Turbinellidae [1]. Structurally, conch is a porcelaneous shell of an oblong or conical form with bulging in the middle and tapering at each end [2]. Conch (*Shankha*) prepared as conch shell ash, known in Ayurvedic literature as *Shankha Bhasma*, is traditionally used in Ayurveda to treat many ailments [3]. According to Ayurveda, conch has cooling (*sheetal*), alkaline (*kshariya*) and adsorbent (*grahi*) properties; has detoxifying (*vishahara*), complexion enhancing (*varnya*) and strengthening (*balya*) actions and is useful for hyperacidity (*amlapitta*), loss of appetite (*agnimandya*), irritable bowel syndrome (*grahani*), pain in the abdomen (*parinaam shool*) and acne vulgaris (*tarunya pidika*) when administered within the maximum therapeutic dose of 250 mg per dose [4]. It is also used internally against dysentery, gonorrhoea, colic, dyspepsia and jaundice [3] in various classical formulations such as *Kaphaketu rasa* (Vol. 1, p. 286), *Chandrodaya Varti* (Vol. 2, p. 193), *Shankha Vati* (Vol. 5, p. 104), *Sutashekhar Rasa* (Vol. 5, p. 371), as one of the major ingredients [5]. Recent research on incinerated conch has focused on pharmaceutical standardization of compressed tablets along with estimation of calcium content and acid neutralization capacity as an antacid [6]. Another comparative clinical study of *Shankha Bhasma*, prepared after purifying with two different methods, was conducted, but no attempt was made to standardize and characterize the incinerated conch. In this clinical study, lemon purified *Shankha Bhasma* revealed significant resolution (*p* < 0.005) of Gastroesophageal Reflux Disease (GERD) symptoms as compared to sour gruel purified *Shankha Bhasma* [7]. Various classical references of *Shankha Bhasma* and its efficacy reports have been reviewed. *Shankha Bhasma* prepared traditionally, and in a muffle furnace, depicted an acid neutralization capacity of 9.5 mE and 7.05 mE, respectively [8]. It also showed in vivo dose dependent protection against gastric ulcer and anti-peroxidative effect without altering serum calcium level, but additional mucin production as compared to standard Ranitidine [9]. It has also shown antispasmodic effect on acetylcholine induced excised rat ileum compared to atropine, therapeutic response to acetic acid induced writhing test in rats and in vitro anti-inflammatory activity related to inhibition of protein denaturation [10]. However, protocol standardization and characterization data which are the basis of further investigations are found to be insufficient in the literature.

Rasashastra is the science of mercury and the branch of Ayurvedic Pharmaceuticals that deals with the preparation of medicines from metals like gold, silver and so forth, and minerals like mica, chalcopyrite and so forth. *Shankha* (Conch) is mentioned in Rasashastra as a mineral under *Sudha Varga* (Calcium group) category [11] and is one of the important Bhasmas used in daily practice by Ayurvedic physicians. *Shankha Bhasma* is generally described to be prepared by soaking the shell in lime juice and calcination in covered crucibles ten to twelve times and finally reducing it to powder [2]. However, the classical texts of Ayurveda describe several methods of incineration of conch using various media like borax [5] (Vol. 5, p. 103), *Citrus limon* juice [12], *Citrus medica* juice [13] (p. 310–311) and Aloe Vera juice [14]. Ayurvedic Formulary of India published by Health and Family Welfare Ministry, Government of India has stated to boil pieces of conch in sour gruel (*Kanjika*) for 3 h and to incinerate 2 times by placing in earthen casserole. Additional use of lemon or aloe vera juice for trituration has also been suggested in these guidelines [15]. Moreover, authoritative books in traditional Unani medicine have mentioned several methods to incinerate conch which includes media like sulphur or *Tribulus terristris* paste or cow milk or *Calotropis procera* latex or lemon juice [16]. Thus, it is clear that various methods of preparation of *Shankha Bhasma*, have been reported. However, the complete pharmaceutical standardization with analytical results except specifications like description, loss on drying, acid insoluble ash, loss on ignition and calcium assay, is not available [17].

The present study was, therefore, undertaken to develop the protocol for preparation of *Shankha Bhasma*. In this method, purification was done using juice of *Citrus limon* (*Nimbuk*) and incineration by the pulp of aloe vera leaf (*Ghrut-kumari*) among all the media suggested. Physico-chemical characterization of replicated batches of the final product was performed using modern methods such as X-ray Diffraction (XRD), Scanning Electron Microscopy (SEM), Thermogravimetric Analysis (TGA), Particle size analysis, Fourier Transform Infra-Red (FTIR) and ICPOES to understand identity, purity and strength of the product. This has established standard operating procedures (SOP) to commercially manufacture *Shankha Bhasma* and developed its complete specifications for Quality Control and Quality Assurance (QCQA).

## 2. Results

### 2.1. Observations During Preparation of Incinerated Conch (Shankha Bhasma)

Generally, steps that are followed to prepare Bhasmas as per the Ayurvedic text include purification of raw material (*Shodhan*) followed by incineration (*Marana*/*Bhasmikaran*). We followed these two important steps in the preparation of incinerated conch (*Shankha Bhasma*) as detailed in materials and methods. The preparation was carried out in three batches in order to test the uniformity of the final product. Various dilutions of lemon juice were attempted during the purification of conch (*Swedana* procedure). Among these, 3.5× dilution with potable water was observed to be suitable to adjust the desired pH 3.5–4. In order to keep the conches immersed into boiling liquid medium for 3 h, various conch to lemon juice ratios (1:3, 1:4 and 1:5 *w*/*v*) were attempted. Among these 1:4 ratio was found to be sufficient to completely immerse and boil conches without exposing them to open air. The temperature was maintained at around 98 °C throughout the experiment. A maximum of 60 g of loss in conch was recorded during the entire purification process.

Following the purification step, the broken pieces of conches (8 kg) were equally distributed in 04 pots which were found to be effective for evenly heating. Equal quantity of aloe-vera juice (8 kg juice for 8 kg purified conches) for the 1st incineration and reduced quantity (5 kg juices for 7.25–7.65 kg of incinerated conch) for the 2nd incineration were found to be necessary for completing the process to get desired characteristics. Depending upon the quantity of aloe-vera juice, dried pellets of conches and reduced quantity of conch, the amount of cow-dung cakes was also reduced from 28 kg to 22.5 kg. The duration of the 1st incineration ranged from 23–25 h and of the 2nd incineration ranged from 17–19 h. These parameters were found to be sufficient to achieve the end point of the process.

**Figure 1:**
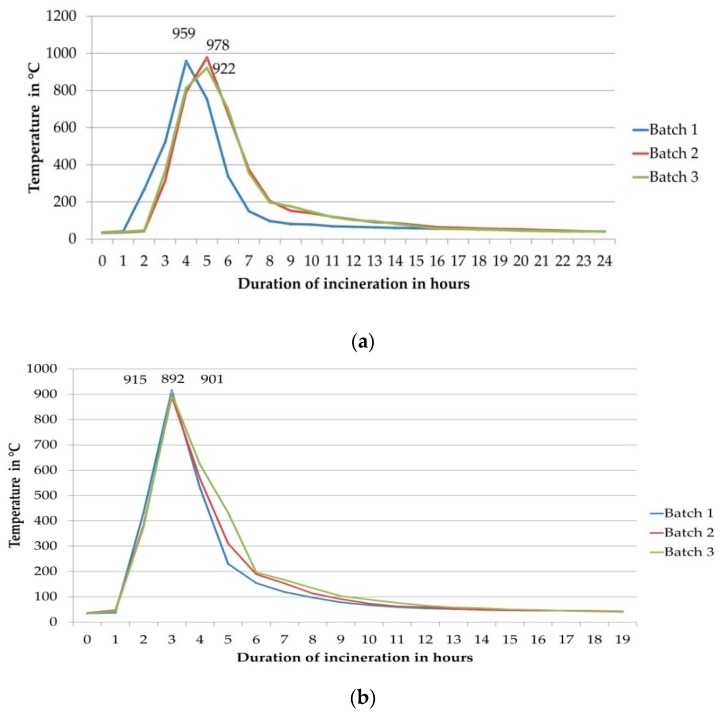
(**a**) Graphical representation of temperature recorded every 1 h during the 1st incineration of Conch. (**b**) Graphical representation of temperature recorded every 1 h during the 2nd incineration of Conch.

During incineration process, temperature was recorded at every 10 min interval. The values at 1 h interval were used for graphical representation of temperature pattern during both the incinerations and are given in Figure 1a,b, respectively. The maximum temperature of three batches was in the range of 922–978 °C during the 1st incineration and 892–915 °C for the 2nd incineration, respectively. The raw data of temperature recorded every hour is given in Supplementary Data Tables S1 and S2. 

This pattern shows that the traditional method of incineration of conch is divided into 3 phases. Phase I is the duration required to attain temperature equivalent to (*T*max—150 °C) from the point of ignition, Phase II is the duration for which the highest temperature range (from ascending and descending *T*max—150 °C) is sustained while Phase III is the self-cooling duration. *T*max is the highest temperature (~900 °C outside the pot) attained during the process and 150 °C is the observed temperature difference between the temperature required to convert aragonite to calcite form of CaCO_3_ (~600 °C inside the pot and ~750 °C outside the pot) and the maximum temperature at which calcite form may get decomposed (~750 °C inside the pot and ~900 °C outside the pot). Table 1 shows the duration and range of temperature values observed for these phases. 

The percentage of yield of conch was used as a measure for pharmaceutical standardization. The stepwise calculation showed that minor loss of not more than 1% *w*/*w* occurred during purification process. Maximum loss of 3.5% to 5.5% *w*/*w* was found after the 1st incineration followed by 2.5 to 3.75% *w*/*w* loss after the 2nd incineration. Finally, raw material to finished product loss was found to be between 6.5% to 10% *w*/*w*, which included loss during grinding and trituration process also. Hence, the final percentage of yield deduced was 90–94% *w*/*w*. The percentage loss at each stage in all the three batches is given in Table 2 while the actual weight measures are enumerated in Appendix A, Table A1.

### 2.2. Analytical Characterization

#### 2.2.1. Analysis of Raw Materials and Processing Media

- Raw conch: The raw conch purchased was identified as animal conch hard material, off white in colour, lustrous, having fishy odour and adhered silt. They were of varying sizes with 10–15 cm in length and 15–20 cm in maximum diameter. The calcium carbonate assay was performed by titration against *di-sodium edetate* [18] and found to be 92.54% *w*/*w*.
- Fresh lemon juice: It was yellow translucent liquid having lemony odour with pH 2.42 ± 0.05 and specific gravity 1.085 ± 0.002.
- Diluted lemon juice: It was whitish yellow translucent liquid having lemony odour with pH 3.58 ± 0.13 and specific gravity 1.054 ± 0.001 gm/mL.
- Fresh Aloe vera (*n* = 7) showed moisture 95.89 ± 0.009% *w*/*w*, total ash 0.87 ± 0.001% *w*/*w*, acid insoluble ash 0.19 ± 0.001% *w*/*w*, alcohol soluble extractive 1.43 ± 0.002% *w*/*w* and water soluble extractive 1.27 ± 0.001% *w*/*w*. The Thin Layer Chromatography of alcoholic extract was run on Silica gel G_60_F_254_ using toluene: ethyl acetate: formic acid: 5:4:1 as a mobile phase. It showed 3 spots at *R*_f_ 0.05, 0.36, 0.83 (all blue) under UV 254 nm, 1 spot at *R*_f_ 0.34 (red) under UV 366 nm and 1 spot at *R*_f_ 0.34 (blue) after spraying anisaldehyde sulphuric acid reagent (Figure not shown).
- Fresh aloe vera juice: Light greenish yellow viscous liquid having pH 4.67 ± 0.13, specific gravity 1.0052 ± 0.0004 gm/mL and viscosity of 3.13 ± 0.39 mP.

#### 2.2.2. Analysis of Purified Conch and Incinerated Conch as per Ayurvedic Classical Tests

- Purified conch: They were milky white, odourless, non-lustrous conch with no deposited silt on it.
- Incinerated conch: Following characteristics of incinerated conch vis-à-vis *Shankha Bhasma* were tested-presented no rubbing or crushing sound when triturated in mortar or crushed between teeth (*Nishabda*), soft in touch (*Shlakshna sparsha*) and entered into the crevices of finger tips (*Rekhapurnatva*), white in colour (*Shweta varna*), tasteless (*Nirasata*), smokeless and odourless when heated (*Nirgandha*) and fine like Kohl (*Anjanopam*). It also passed 100% through mesh of size #200 (75 microns) [19].

#### 2.2.3. Physical and Chemical Analysis of Incinerated CONCH 

Incinerated conch was light greyish white fine powder insoluble in water and soluble in hydrochloric acid with effervescence. It showed 0.19 ± 0.10% *w*/*w* loss on drying and pH 9.96 ± 0.361% of 1% solution in distilled water. The bulk and tapped density was 0.773 ± 0.036 and 1.287 ± 0.081, respectively. The calcium (Ca as CaCO_3_) assay was done by titration against *di-sodium edetate* and calcium percentage was found to be 39.05 ± 0.36% *w*/*w* [18].

### 2.3. Characterization of Conch Using Modern Analytical Techniques

#### 2.3.1. X-Ray Diffraction (XRD) Analysis

**Figure 2:**
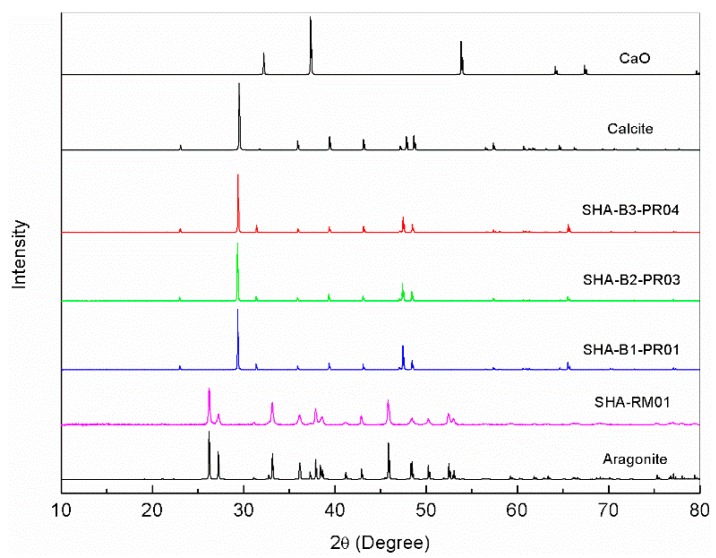
XRD pattern of incinerated conch simulated with Aragonite, Calcite and calcium oxide.

Raw powdered conch and three batches of incinerated conch were analysed by powder X-Ray diffraction. Comparative simulated XRD patterns of aragonite and calcite forms of calcium carbonate as well as that of calcium oxide are shown in Figure 2. In this figure, SHA-RM01 denotes crude conch while incinerated conch of batches 1, 2 and 3 are represented by SHA-B1-PR01, SHA-B2-PR03 and SHA-B3-PR04, respectively. A direct comparison of the XRD patterns of the conch samples with the simulated patterns shows that the raw conch is calcium carbonate only in the aragonite form without any calcite content. On the other hand, the three batches of the incinerated conch reveal the presence of only calcite form of calcium carbonate without any aragonite content. Calcium oxide has not been detected in both, the raw and the incinerated conch samples.

#### 2.3.2. Scanning Electron Microscopy (SEM) analysis

**Figure 3:**
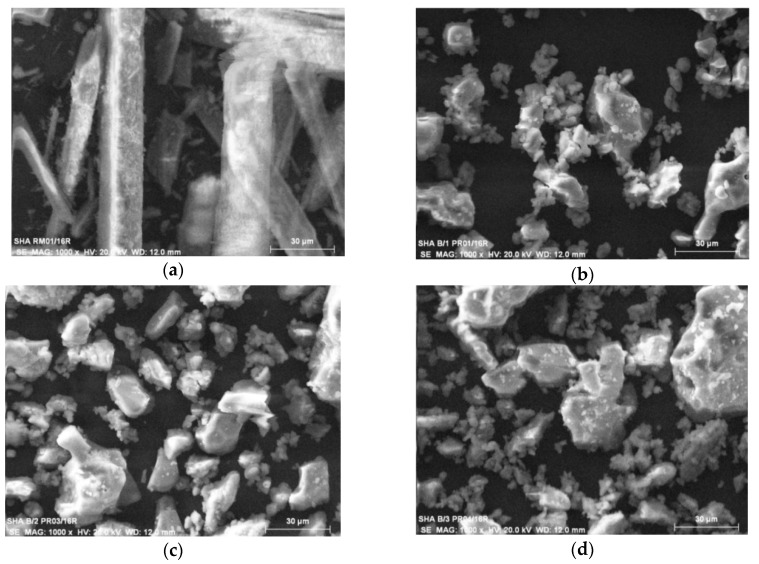
Scanning Electron Microscopy of raw and incinerated conch at magnification 1000× (Scale: 30 µm) (**a**) Powdered raw conch; (**b**) Incinerated conch-Batch 1; (**c**) Incinerated conch-Batch 2; (**d**) Incinerated conch-Batch 3.

Both raw and incinerated conch was examined under Leica Cambridge 440 SEM (Leica Cambridge Ltd., Cambridge, UK) to elucidate the structure and particle size. The raw and incinerated conch showed distinct pattern while the three batches of incinerated conch revealed uniform patterns at all the magnifications under study. A representative figure of 1000× magnification for raw conch (Figure 3a) and the three batches of incinerated conch (Figure 3b–d) are given below. The raw conch showed rod like structures of breadth 12–20 micron range while the incinerated conch revealed uniform polygonal structure in the range 6–30 micron. Moreover, the same particle range of all the three batches indicated uniformity of the final product.

#### 2.3.3. Particle Size Analysis

**Figure 4:**
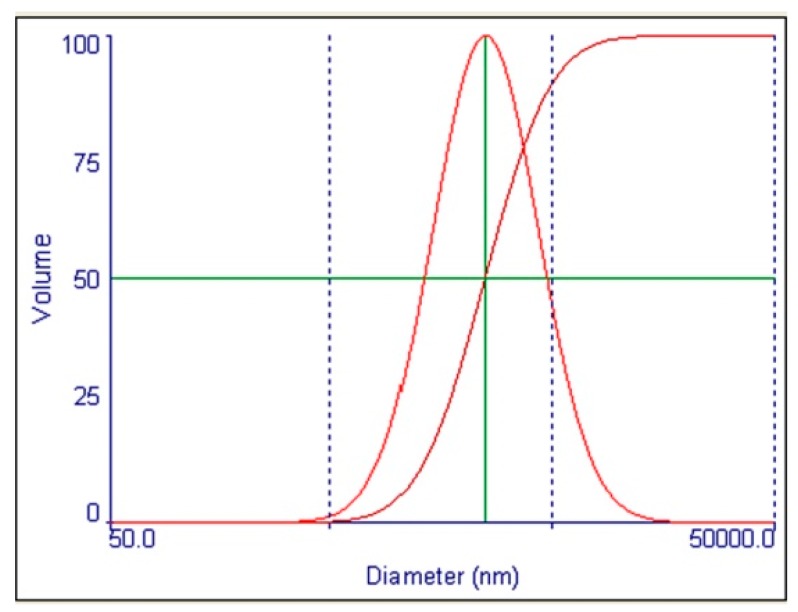
Particle size distribution of finished product - incinerated conch (*Shankha Bhasma*).

Incinerated conch as a finished product, after grinding, showed effective diameter ranging from 400–750 nm, mean diameter from 200–350 nm and poly-dispersity from 0.0100–0.0600. The particle distribution of *Shankha Bhasma* samples showed that 10% particles were below 1200 nm, 50% particles below 2600 nm and 90% particles below 5500 nm. The representative profile of one of the batches is given as Figure 4.

#### 2.3.4. Thermogravimetric Analysis (TGA)

**Figure 5:**
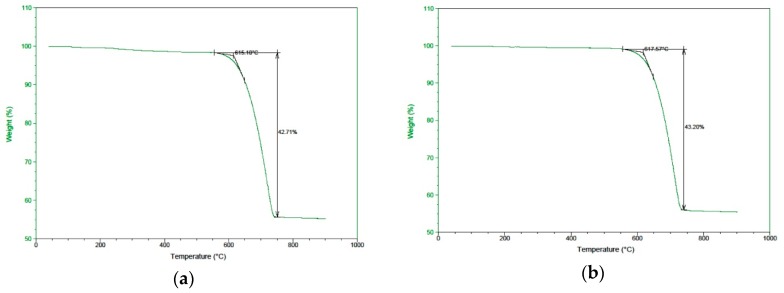

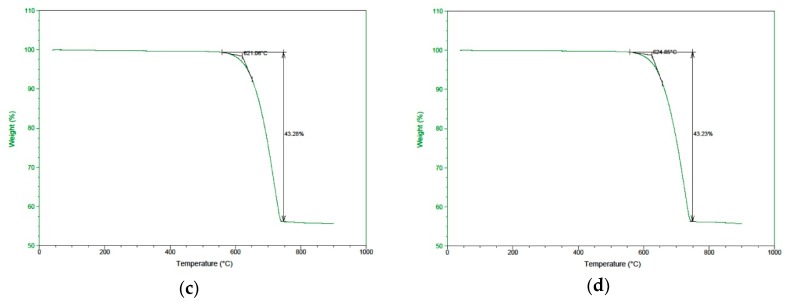
Graphical presentation of Thermogravimetric analysis of raw and incinerated conch showing percent weight loss against temperature (°C): (**a**) Raw conch; (**b**) Incinerated conch-Batch 1; (**c**) Incinerated conch-Batch 2; (**d**) Incinerated conch-Batch 2.

TGA was performed for raw conch and all the three batches of incinerated conch on TGA Q5000 (TA Instruments, New Castle, DE, USA) and the temperature and percentage loss in samples were noted. Sharp drop in weight (Figure 5a) initiated at 615.10 °C for raw conch and 612.44 ± 2.10 °C for three batches of incinerated conch was observed, respectively (Figure 5b–d). Raw conch showed weight loss of 42.71% between 615.10 to 750 °C while batch 1, 2 and 3 of incinerated conch depicted weight loss of 43.20, 43.28 and 43.23% against temperature 740, 750 and 750 °C, respectively. Total weight loss of 42.71% and 43.23 ± 0.02% was observed for raw conch and incinerated conch after 750 and 746.67 ± 3.33 °C, respectively. Raw conch and the incinerated conch remained stable beyond 750 and 746.67 ± 3.33 °C, respectively upto 1000 °C. The stage-wise loss of moisture content (100 °C), organic matrix (450 °C) and CO_2_ (upto 850 °C) is detailed in Appendix A, Table A2.

#### 2.3.5. Fourier Transform Infra-Red (FT-IR) Analysis

**Figure 6:**
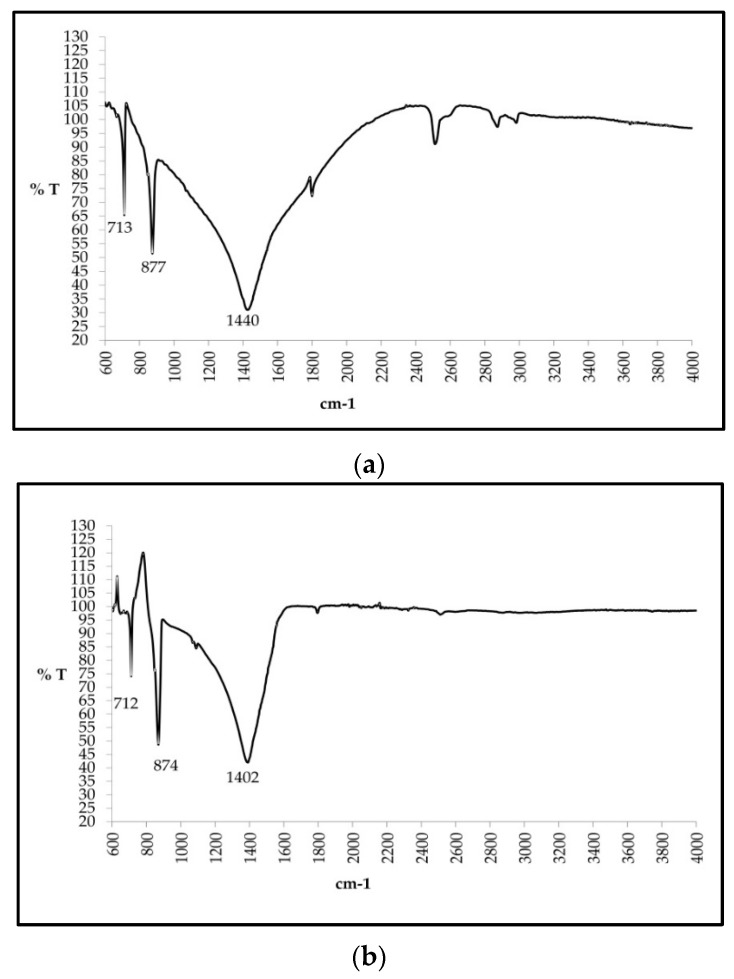

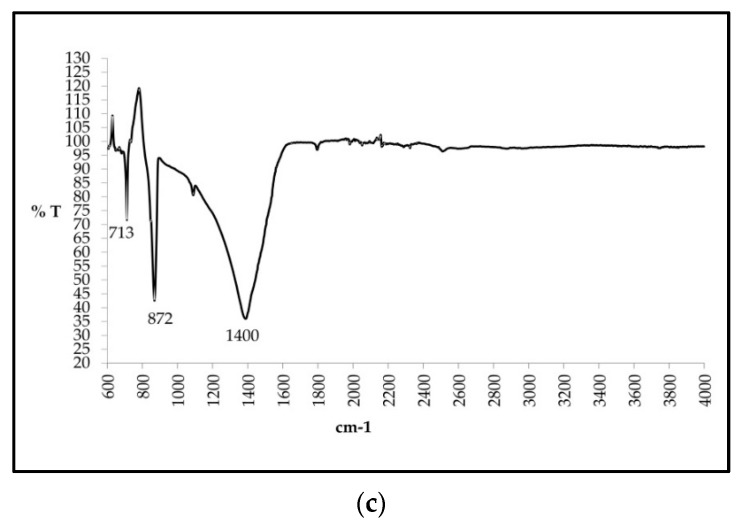
Fourier Transform Infrared Analysis of incinerated conch showing CO_3_^2−^ vibrations: (**a**) Incinerated conch-Batch 1; (**b**) Incinerated conch-Batch 2; (**c**) Incinerated conch-Batch 3.

All the three batches of incinerated conch were analysed on FTIR (Perkin Elmer Inc., Waltham, MA, USA). Batch 1 of incinerated conch showed peaks at wave-lengths 1440, 877 and 713, Batch 2 at 1402, 874 and 712 and Batch 3 at 1400, 872 and 713, respectively as shown in Figure 6a–c. All these wavenumbers of all the three batches showed common characteristic peaks which are more similar to symmetric and asymmetric CO_3_^2−^ vibrations.

#### 2.3.6. Inductively Coupled Plasma Optical Emission Spectrometry (ICPOES)

Raw material and all the three batches were analysed on ICPOES for Calcium, Magnesium and heavy metals (Hg, As, Cd and Pb). The wavelengths showing repeatable and highest values of intensities were selected for calculations (Ca: 393.366 nm, Mg: 279.553 nm, Hg: 184.959 nm, As: 189.042 nm, Cd: 228.802 nm and Pb: 220.353 nm). The analysis showed that raw conch contained 41.45% *w*/*w* Ca and 0.020% *w*/*w* Mg. Finished products showed 39.29% *w*/*w*, 40.39% *w*/*w*, 39.75% *w*/*w* of Ca in batch 1, 2 and 3, respectively. The percentage of Mg was found to be 0.040% *w*/*w*, 0.034% *w*/*w* and 0.033% *w*/*w* in batch 1, 2 and 3, respectively. Calculated average calcium and magnesium in *Shankha Bhasma* was 39.81 ± 0.319% *w*/*w* and 0.035 ± 0.0022% *w*/*w*, respectively.

In heavy metal analysis, Hg and Cd were not detected in both, raw conch as well as the three batches of incinerated conch. Arsenic (As) was not detected in raw conch, batch 2 and batch 3 of finished product, whereas 1.61 ppm was detected in batch 1. The concentration of lead (Pb) was found to be 6.69 ppm in raw conch and 1.72 ppm in batch 1 whereas it was not detected in batch 2 and 3 as given in Table 3.

### 2.4. Monograph of Incinerated Conch (Shankha Bhasma)

Based on the results obtained using manufacturing methods and analytical techniques, the monograph was developed for 8 Kg of commercial batch (*n* = 3 batches). Table 4 and Table 5 show their parameters and specifications.

## 3. Discussion

Incinerated conch (*Shankha Bhasma*) is one of the important medicines used in various Ayurvedic formulations. The characteristics of Molluscs are described as outer horny layer, a median prismatic layer of lime salts and an inner pearly nacreous layer. It is greatly variable in shape, structure and colour, forming an outstanding external feature in the animal [20]. Being a natural marine animal origin product, there are several forms of conches found in nature. In Indian market, conch is available in three forms viz., whole, central stalk and pieces (personal observation). As per the *Rasashastra* literature, conch which is whole, white in colour, shining and heavy in weight, is recommended for Ayurvedic use [4] and hence whole form of conch was used in the present study. Raw conch is animal conch that contained 90% of Calcium as Calcium carbonate estimated by titration and XRD (Figure 2) while rest being organic matrix, soil and moisture. 

*Shankha* is sold without cleaning; hence it was washed after soaking in hot water. This helps to remove the physical impurities like sand deposits. Further any sour liquid is recommended for complete purification of conches [21]. Also sour media is mentioned to be used for purification of metals and minerals [13] (p. 158). Lemon (*Citrus limon*) was selected as purification medium being readily available. The fresh juice of lemons is highly acidic (pH 2–2.5), so when poured on conch readily reacts with calcium carbonate, thus forming citrates and evolution of carbon dioxide. Hence, to avoid this, fresh juice was diluted to adjust pH, so that only impurities like organic matter on surface of conch would get eliminated. They were again thoroughly washed with hot water and dried for further use. This made the conch clean, bright white coloured, with no smell and lustre.

Fresh aloe vera pulp used for trituration had moisture not less than 98% *w*/*w*, pH 4.25–5, specific gravity 1.0040–1.0060 g/cc and viscosity from 2−4 mP as reported earlier [22,23]. It contains an inner clear gel like pulp with 98.5% water and the remaining with glucomannans, amino acids, lipids, sterols, vitamins [24] and polysaccharides [25]. Thus, aloe vera fresh juice helped to wet grind the calcium carbonate particles smoothly, bind them easily during pellets formation and got dried in a short duration without leaving substantial inorganic residue after incineration. Most importantly, modern pharmaceutical industry has been using biopolymers like chitosan, alginate or K*-*carrageenan [26] and xanthan, sodium alginate and pectin [27] as substrate to contribute crystallization of calcium carbonate. Aloe vera pulp rich in polysaccharides may have similar function of biopolymer during incineration of conch. Role of aloe vera juice as a triturating medium has been studied in another *Sudha varga* marine drug called *Shouktik* (Mother pearl) by incinerating the material triturated with and without aloe vera juice. It was found that trituration prevents formation of CaO by interfering with heat transfer and maintaining the product in calcite form. Additionally, it helps to reconvert Ca(OH)_2_ to CaCO_3_ by maintaining CO_2_ atmosphere due to burning of organic matter in aloe vera juice [28]. Time dependent XRD and SEM analysis could have confirmed this observation in the present study also. However, arresting of the reaction at various time points was not possible in the current protocol.

The traditional texts have only mentioned the use of conch as raw material, aloe vera as liquid media and cow-dung cakes for incineration. However, no quantitative data for any of these steps are available in the literature. Hence, the amount of conch (in kg), liquid media (in L), quantity of cow-dung cakes (in kg) and number of earthen pots (count) were standardized. In this study, the size and quantity of crushed conches and liquid media quantity were taken into consideration while fixing the quantity of cow-dung cakes during the 1st incineration, after conducting pilot experiments. The main objective of the 1st incineration was to burn the organic matter and aloe vera juice as detailed in Material and Method section. During the 2nd incineration, the traditional sequence was followed as shown in Figure 6. During second incineration, the quantity of cow-dung cakes was reduced to avoid overheating leading to conversion of calcium carbonate to calcium oxide. The observed value 39.05% of calcium as calcium carbonate (Molecular Weight 100.42 g/mole) determined by titration in present study is in accordance with the theoretical value of 40% rather than the reported values 44–46% [29] and 48.6% [30] of incinerated conch. Moreover, as reported previously [31] similar method of incineration but without adding aloe vera juice was attempted, which showed incinerated conch as calcium oxide. However, incinerated conch (*Shankha Bhasma*) does not give pungent taste character as shown by calcium oxide. Thus, it is necessary to have appropriate calcium carbonate form in *Shankha Bhasma* rather than calcium oxide [32].

The most critical step of synthesis of *Shankha Bhasma* is the incineration step. Three Phases during both the incinerations observed in our studies are detailed in Table 2. The temperature and its pattern were needed and sufficient to transform aragonite structure of calcium carbonate to calcite form. The temperature attained inside the pots initially evaporated water and evolved gases from the breakdown of organic matrix. This makes the aragonite free from extraneous matter and makes it easy to rearrange itself to calcite form. The first incineration was more intended to break down the layered structure, destruction of organic matrix and water evaporation followed by conversion of aragonite to calcite form. The second incineration was required for conversion of remaining aragonite form to calcite and its stability by avoiding overheating beyond 750 °C. The highest temperature attained outside earthen pot during the 1st incineration was around 900–1000 °C and during the 2nd incineration 800–900 °C. However, the inner temperature can be assumed to be between 700–800 °C for the 1st incineration and 650–750 °C for the 2nd incineration, the pots being of earthen nature. This is in conformity with other studies conducted on aragonite to calcite transformation [33]. In this reported study, the structural and mechanical stability of conch shell was studied at 310, 500 and 900 °C. At 310 °C, the low content biopolymer burned out easily, phase transformation from aragonite to calcite look place at 500 °C while calcite to lime conversion was observed at 900 °C which induced structural modification and deteriorated mechanical stability, respectively.

To validate these observations, various analytical tools viz., XRD, FTIR, TGA and SEM were employed in the present study.

The XRD pattern of both raw and finished conch (Figure 2) when simulated with standards, clearly indicated that raw conch is aragonite in nature, while after incineration this aragonite structure got rearranged to calcite form. Moreover, absence of any other major peaks indicated that incinerated conch was in pure calcite form. Previous X ray diffraction studies of aragonite crystal showed that aragonite was successfully transformed to calcite by heating above 488 °C [34]. It has been reported that calcium in calcium carbonate calcite form is better absorbed as compared to its aragonite form [35]. Furthermore, FTIR analysis of incinerated conch (Figure 5) showed common characteristic peaks present at wavenumbers 1410, 874 and 712 cm^−1^ which are more similar to CO_3_^2−^ vibrations (symmetric and asymmetric) and are comparable with recent researches on calcium carbonate nanoparticles [36]. 

This was further confirmed by TGA analysis of raw and incinerated conch (Figure 4). Conch is a biogenic aragonite. For the biogenic sample the effect of thermal treatment is that, it reduces the intrinsic strain through the degradation of the macromolecules forming the organic matrix of the nacre [37]. The TGA curve in our study showed negligible (1–2% *w*/*w*) weight loss till 600 °C. Previous DTA-TG study conducted on several aragonites clarified the transformation of aragonite to calcite. It has concluded that biogenic aragonites contain flowing water upto 0.1–1.3% which evaporates during heating along with CO_2_ evolved from the combustion of other organic material. The study showed that water seems to play significant role of making some hydrothermal conditions, where calcite nucleation occurs thus triggering the transformation [38]. In our method of *Shankha Bhasma* preparation, conch is incinerated with aloe vera pulp as an added biopolymer for complete conversion of aragonite to calcite. A further significant weight loss with higher slope between 600–750 °C was recorded in our study. This weight loss ranged from 42% to 43% *w*/*w* which indicated decomposition of calcite to calcium oxide and CO_2_. When heated above 800 °C, profile remained stable up to 1000 °C. This was in accordance with the previous findings of TGA analysis of Calcite [39,40].

SEM analysis of raw conch and finished product (Figure 3) also confirmed the structural transformation of aragonite to calcite. The rhomboidal structure of calcite and a needle like shape of aragonite was found as expected morphologies [41].

The particle size distribution study showed the range. The values are unique for this method of preparation and may help for quality control and quality assurance. Calcium carbonate nanoparticles have wide range of applications in drug delivery system due to its unique properties like accessibility, low cost, safety, biocompatibility, pH sensitive properties and slow biodegradability [42].

As per quality parameters of Ayurvedic Pharmacopoeia of India, elemental Ca is reported to be in the range of 38–40% *w*/*w* while heavy metals need to be within the permissible limits (Hg: 1 ppm, As: 3 ppm, Cd: 0.3 ppm and Pb: 10 ppm) in the finished product of conch [15,17]. It has also been reported to have presence of Mg as trace element (<1.5% *w*/*w*) in the incinerated conch [32,43]. In the present study, conventional titration method detected elemental Ca as 39.02% *w*/*w* keeping in line with the pharmacopoeia guidelines. Moreover, an additional sensitive method of ICPOES used in the present study revealed simultaneous and precise estimation of Ca, Mg and heavy metals to be within the permissible limits in both, raw and incinerated conch (Table 3).

All these experimental studies conducted through advanced instruments are helpful to understand the pharmaceutical transformation of aragonite conch into better absorbed calcite known as *Shankha Bhasma* in Ayurveda. Rasashastra texts have mentioned *Shuddha Shankha*/purified conch (aragonite form) for external applications as coryllium in eye diseases and Shankha Bhasma/incinerated conch (calcite form) for internal use in several diseases ([4], pp. 285–289). *Shankha Bhasma* being a traditional drug, the authors is clinically investigating the role of *Shankha Bhasma* as adjunct treatment in GI malignancies, mainly stomach and colo-rectal cancers, by assessing the GI symptoms like nausea, vomiting, anorexia, flatulence, indigestion, mucositis, hyperacidity caused due to these malignancies as well as toxicities of chemotherapy. Further experimental studies to reveal the pharmacodynamics and pharmacokinetics of incinerated conch can be designed using this preliminary data in the form of SOP to manufacture quality drug.

## 4. Materials and Methods

### 4.1. Manufacturing Process

#### 4.1.1. Procurements

Raw conch was purchased from Agricultural Produce Market Committee, Vashi, Navi Mumbai (MS), India. Selection of conch was made based on the characteristics described in classical texts such as colour, structure and weight [4]. Lemon fruits (*Nimbuk*) were purchased from Mahatma Phule vegetable market, Pune (MS), India whereas Aloe vera (*Ghrut-kumari*) was collected from the Sanjeevani Plantation Unit of Bharatiya Sanskriti Darshan Trust, Wagholi, Pune (MS), India. Lemon was identified as *Citrus limon* (Linn.) Burm. Syn. *Citrus medica* var *limonum* and aloe vera as *Aloe barbadensis* mill. Syn. *Aloe vera* at Indian Drug Research Association and Laboratory, Pune (MS), India.

#### 4.1.2. Purification of Conch (*Shankha Shodhan*)

1.  Pre-procedure

Three batches of raw conch (8 kg each) were immersed in hot potable water overnight; surface was cleaned with soft brush to remove silt and was finally dried under shade. Lemons were cut and compressed to extract fresh juice. It was further diluted with 4 parts of potable water. 

2.  Main procedure

Assembly of traditional boiling apparatus (*Dolayantra*): Washed conches were kept on cotton cloth of 1 m × 1 m and placed hanging in Stainless Steel (304) vessel, 50 L capacity with the help of 30 cm × 1 cm (LXD) SS rod. The conches were then immersed completely in diluted lemon juice and boiled for 3 h. Temperature was maintained and recorded throughout the experiment.

3.  Post-procedure

After 3 h, conches were removed, washed with lukewarm water to clean them properly and dried in shade till further use. All the details of the purification process are given in Table 6. 

#### 4.1.3. Incineration of Conch (*Marana of Shankha*)

**Figure 7:**
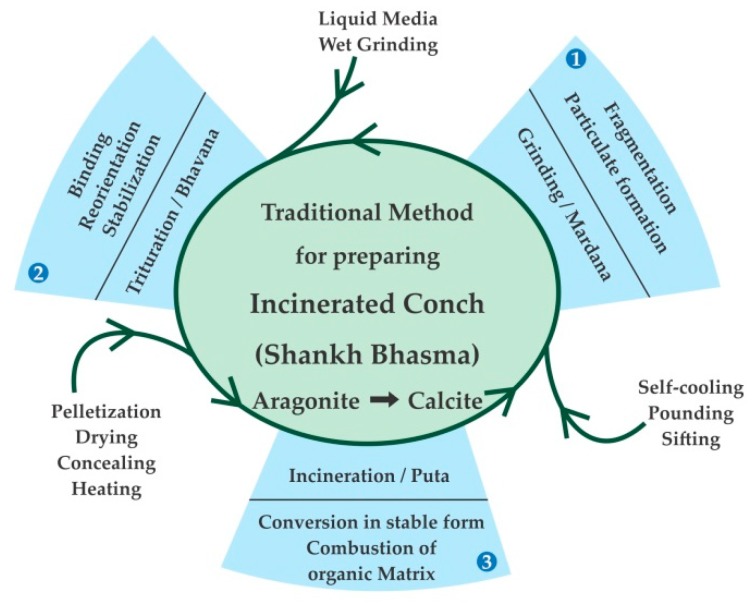
Schematic presentation of incineration of conch and probable role of each step involved.

Figure 7 depicts the schematic presentation of incineration processes including three major steps with intermediate processes and probable role of each step to manufacture incinerated conch (*Shankha Bhasma*). The process was initiated with grinding (*Mardana*) of purified conch followed by trituration (*Bhavana*) with aloe vera pulp and incineration (*Puta*). These were repeated till the desired characteristics were achieved.

1.  Pre procedures

Extraction of Aloe vera pulp: The fresh leaves were cut to exclude tip, base and side thorny ridges. The leaf was then cut half through the pulp. The pulp was scrapped as lumps and ectoderm was discarded. The pulp in lump form was homogenized in juicer and strained through mesh #22 for further use.

**Figure 8:**
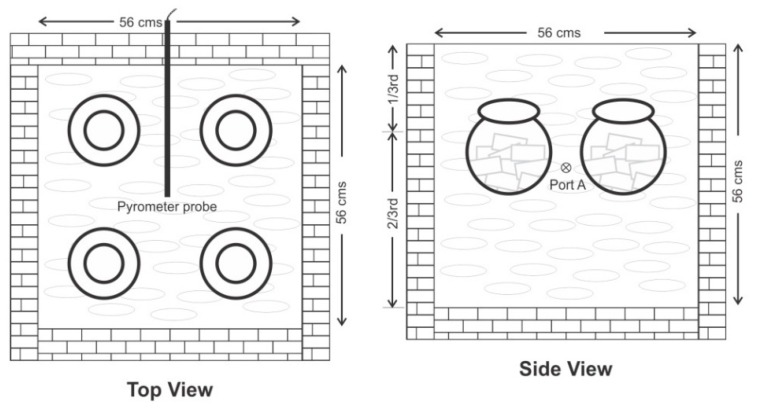
Top view showing placement of pots during incineration and Side view showing position of pyrometers along with distribution pattern of cow-dung cakes placed.

2.  Main procedure

- Pounding and Grinding (*Mardana)*: Purified conches were fragmented into small pieces of approximately 2 cm × 2 cm × 2 cm by pounding in a 2 L capacity cylindrical pounding instrument made up of cast iron before incineration. After 1st incineration the material was powdered using pounding instrument and electrical mixer grinder of 1200 Watts motor having 2 litre capacity vessel with lid. Sifting was done by 40# SS sieve of 18inch diameter made up of 304 SS attached to Vibro-sifter. After 2nd incineration, pounding/ grinding step was repeated as above and sifting was done by 200# mesh.
- Trituration (*Bhavana*): During 1st trituration, aloe vera juice was added to fragmented conch in equal proportion, mixed thoroughly and incinerated directly. During 2nd trituration, electrically operated wet grinder of 10 L capacity vessel (45 cm Height and 35 cm Diameter) was used for trituration. The granite double rollers of the wet grinder were attached to a central shaft. All contact parts were of 304 SS or granite stone. Sufficient quantity of aloe vera juice was added to ground conches to make slurry and to wet grind for 3 h. The quantity of aloe vera juice used in both the trituration processes was noted.
- Pellet formation (*Chakrika*) and Drying (*Shoshan*): Trays made up of 304 SS having 2.5 cm Height, 45 cm Width and 90 cm Length were used for spreading 2 Kg of wet slurry of triturated conch per tray. The thick paste was cut in crisscross fashion with a knife to get uniform pellets of 2.5 cm × 2.5 cm size. The pellets were dried at 105 °C for 3 h in Industrial tray dryer with moisture exhaust facility.
- Concealing in earthen vessels (*Sharava samputa*): The pellets (2 Kg per pot) were placed in clean round 2 litres capacity earthen pots having 4 inch mouth affixing earthen lids. Earthen vessels were concealed with China clay of mesh size 80, smeared on cotton cloth of 3 inch width and 1 meter length. The concealed pots were dried in shade before subjecting to incineration.
  Incineration arrangement (*Puta*): The traditional method of incineration in a furnace called *Gajaputa* (*Gaja* = Elephant), that is, the space required for a baby elephant to sit (56 cm × 56 cm × 56 cm) is indicated for the incineration of conch [4,13]. In this experiment, fire bricks were used to construct *Gajaputa* in a closed 37 Square meter. room. Four pots diagonally and laterally equidistant were placed in the heap of cow-dung cakes in the incinerator (Figure 8 Top view). Cow-dung cakes (moisture content of 12 ± 5% *w*/*w* and ash value of 28 ± 5% *w*/*w*) taken on weight basis for each incineration were placed as 2/3rd below and 1/3rd above the earthen vessels containing conches. Specific arrangements for 69 cm long sensor Pyrometers with Data Logger System (PPI Analytics make, RT to 1200 °C, Digital display with Universal Serial Bus port) were made. A pyrometer probe was centrally placed in each pit as shown in the schematic presentation of pyrometer (Figure 8 Top and Side view). The arrangement was ignited at the four corners of pit and allowed to self-cool without disturbing the arrangement. The temperature recording in the data logger system was set at 10mins interval and the values were retrieved through USB port.

All the quantitative details of aloe vera juice, cow-dung cakes and batch sizes of all the three batches is summarized in Table 7.

### 4.2. Storage Conditions

Liquid media required for purification and trituration experiments were stored in amber coloured Poly Ethylene Terephthalate bottles in refrigerator at 4 °C while dry materials in polyethylene bags in cool and dry place till further use. Finished product was stored in High Density Poly Ethylene bottles, foil sealed and labelled.

### 4.3. Characterization of Raw Materials and Finished Product

#### 4.3.1. Analytical Characterization

Organoleptic properties [4] and calcium carbonate [19] assay of raw conch were carried out as per the standard protocols. Organoleptic parameters and physical evaluation were carried out for purified conch. Specific gravity and pH were measured for lemon and aloe vera juice (IP, 2007) while viscosity was noted for aloe vera juice alone. Raw aloe vera leaves were also tested for moisture content, ash value, acid insoluble ash, water soluble extractive value, alcohol soluble extractive value and Thin Layer Chromatography as basic parameters. Classical Ayurvedic parameters such as sound, touch, colour, taste, odour and fineness were evaluated for incinerated conch. Similarly, loss on drying, pH, bulk and tapped density [44] and calcium assay were also performed for incinerated conch [19]. 

#### 4.3.2. Physico-Chemical Characterization

- X-Ray Diffraction: Raw powdered conch and 3 batches of incinerated conch were analysed on PANalytical X’PERT Pro X-Ray (Lelyweg, Almelo, Germany) Diffractometer using Cu Kα radiation (λ = 1.5418 Å). XRD patterns were recorded in the 2θ range 10–80° at a scanning rate of ~2°/min. Powder XRD patterns of aragonite, calcite and calcium oxide were simulated from the known crystal structure parameters of these compounds reported in the literature, using the a free software ‘Powder Cell for Windows’ (Version 2.4, Federal Institute for Materials Research and Testing, Berlin, Germany available at http://www.ccp14.ac.uk) and compared with the experimental patterns. Phase content was analysed by least squares refinement of the experimental patterns. The error in the phase content analysis, if more than one phase was present, was ±10%.
- Scanning Electron Microscopy: Scanning electron microscopy (SEM) analysis was carried out on a Leica Cambridge 440 scanning electron microscope. The crude conch was powdered in iron mortar and sieved through 200# mesh (75 microns) and incinerated conch was used as is. Samples were prepared by spreading the powder sample on a double-sided carbon tape and sputter coated with gold for nullifying the charging effect. All samples were observed with various magnifications ranging from 100×, 500×, 1000×, 2000× and 5000×.
- Thermogravimetric Analysis: The TGA analysis was done using TGA Q5000 (hanging pan machine) from TA Instruments Inc., New Castle, DE, USA. The measurements were done under nitrogen atmosphere and the temperature range used was 40–900 °C with a heating rate of 10 °C/min. For each measurement about 5–10 mg sample was used. Data analysis was performed using Universal analysis software.
- Fourier Transform Infra-Red spectroscopy: All the three batches of incinerated conch were analysed on FTIR (Model: Spectrum GX, Perkin Elmer, Waltham, MA, USA) under Attenuated Total Reflection (ATR) mode with scanning range in mid IR from wavenumber 4000–600 cm^−1^. Number of scans recorded was 10 in % Transmission mode with resolution 4.
- Particle size analysis: The particle size measurements were performed with the dynamic light scattering (DLS) technique using 90 Plus instrument from Brookhaven Instruments Corp., Holtsville, NY, USA equipped with a 35 mW solid state laser and a highly sensitive avalanche photo diode (APD) detector. Incinerated conch (*Shankha Bhasma*) powder was suspended in filtered milliQ water and diluted to avoid the effect of multiple scattering. The measurements were done at an angle of 90 degrees for a period of 3 min with 3 technical replicates for all three batches. The particle size was obtained from the intensity autocorrelation function data. From the light scattering data, volume weighted particle size distribution was obtained and D10, D50 and D90 were calculated. The span of the distribution was calculated using the formula, SPAN = (D90 − D10)/D50.
- Elemental Assay by Inductively Coupled Plasma Optical Emission Spectrometer (ICPOES): Both raw and incinerated conch were analysed and quantified for Calcium (Ca), Magnesium (Mg) and heavy metals (Hg, As, Cd, Pb) using ICPOES (Thermo Fisher Scientific, Waltham, MA, USA) by following methodology established in our laboratory. The samples were closed digested in Ethos Easy Advanced Microwave Digestion System (Milestone SRL, Milan, Italy) for heavy metals and open digested for Ca and Mg. In closed method, 200 mg sample and 6 mL Conc. HNO_3_ were placed in the microwave vessel and digested gradually for 15 min to achieve 200 °C temperature, then maintained for 20 min and finally cooled to room temperature. In open method, 100 mg sample and 2% HNO_3_ acid were allowed to react in a Tarson centrifuge tube. Standards for Ca, Mg, As, Cd and Pb were from Merck (Certipur, Darmstadt, Hesse, Germany) and that of Hg from Sigma Aldrich (TraceCERT, St. Louis, MO, USA). Standard solution of 1000 ppm was diluted to 1, 2, 4 and 6 ppm for Ca and Mg whereas 5, 10, 20 and 50 ppb for heavy metals. Digested samples of raw and incinerated conch were made up to 50 mL in MilliQ water for as stock. For final analysis, 250 μL and 5 mL stock solutions were diluted to 50 mL for Ca and Mg, respectively while stock for heavy metals was used as it is. The final samples were aspirated in Argon gas flame in iCAP7200 Duo ICPOES system with auxiliary gas flow of 0.5 L/min. The intensities were measured at wavelengths 393.366 and 396.842 for Ca, 279.553 and 280.270 for Mg, 184.959, 194.227 and 253.652 for Hg, 189.042, 193.759 and 449.423 for As, 228.802 and 226.502 for Cd and 220.353 and 216.999 for Pb. The wavelengths exhibiting R^2^ value more than 0.999 were selected for calculation of the respective elements. Ca and Mg were measured in radial view while heavy metals in axial view. The calibration graph for standards was plotted using Qtegra Software Version 2.6 (Thermo Fisher Scientific, Waltham, MA, USA) and final concentration was measured as the percentage of each element using the dilution factor.

## 5. Conclusions

Natural marine resources like conch when processed meticulously as per the Ayurvedic classical texts can be transformed to calcite form of Calcium carbonate. The Standard Operating Procedure laid here can be followed to get batch to batch consistency. Modern theories and techniques can be used to understand the pharmaceutics of Ayurvedic methods of preparation of medicines. Incinerated conch being one of the important therapeutic agents in Ayurveda, if not prepared appropriately may not show desired clinical effects. The monograph thus developed may be helpful for quality control. 

## Figures and Tables

**Table 1:** Phases of temperature range during each incineration of conch in three batches.

Phase	Batch 1		Batch 2		Batch 3	
	D (H:Min)	*T* °C	D (H:Min)	*T* °C	D (H:Min)	*T* °C
Incineration 1						
I	4:40	36–782	4:10	33–818	4:00	35–813
II	1:10	783–959–794	1:20	819–978–857	1:50	814–922–789
III	16:10	793–41	16:30	856–45	16:10	788–41
Incineration 2						
I	2:40	34–756	2:40	36–746	2:40	34–755
II	0:50	757–915–789	0:50	747–892–766	0:50	756–901–752
III	15:30	788–41	15:30	765–42	15:30	751–40

**Table 2:** Percentage of yield and loss of incinerated conch through various stages in all the three batches.

Steps	Batch 1		Batch 2		Batch 3	
	% Yield	% Loss	% Yield	% Loss	% Yield	% Loss
Raw conch–Purified conch	99.37	0.63	99.50	0.50	99.25	0.75
Purified–1st incineration	94.52	5.48	96.11	3.89	95.47	4.53
1st incineration–2nd incineration	96.48	3.52	97.39	2.61	97.10	2.90
2nd incineration–Finished product	99.32	0.68	99.33	0.67	99.46	0.54
Raw–Finished	90.00	10.00	93.13	6.87	92.00	8.00

%: Percentage calculated on the basis of weight records.

**Table 3:** Details of ICPOES analysis of raw and incinerated conch (Batch 1, 2 and 3).

Steps	Ca		Mg		Hg		As		Cd		Pb	
**Raw Conch**												
Weight (g)	0.109	0.101	0.109	0.101	0.101	0.104	0.101	0.104	0.101	0.104	0.101	0.104
Reading	4.772	3.988	0.038	0.046	−61.73	−64.55	−2.013	−0.155	−1.636	−1.316	13.145	14.46
Result	43.42	39.49	0.017	0.023	ND	ND	ND	ND	ND	ND	6.47	6.91
Mean	41.45		0.020		ND		ND		ND		6.69	
**Batch 1**												
Weight (g)	0.101	0.101	0.101	0.101	0.101	0.100	0.101	0.100	0.101	0.100	0.101	0.100
Reading	4.043	3.936	0.080	0.083	−67.86	−82.26	3.72	2.795	−1.582	−1.592	3.835	3.119
Result	39.72	38.74	0.039	0.041	ND	ND	1.84	1.39	ND	ND	1.90	1.55
Mean	39.23		0.040		ND		1.61		ND		1.72	
**Batch 2**												
Weight (g)	0.105	0.100	0.105	0.100	0.102	0.101	0.102	0.101	0.102	0.101	0.102	0.101
Reading	4.211	4.100	0.075	0.064	−4.017	−3.836	−1.062	−4.162	−2.081	−1.9	−6.568	−8.171
Result	39.99	40.80	0.036	0.032	ND	ND	ND	ND	ND	ND	ND	ND
Mean	40.39		0.034		ND		ND		ND		ND	
**Batch 3**												
Weight (g)	0.102	0.106	0.102	0.106	0.103	0.105	0.103	0.105	0.103	0.105	0.103	0.105
Reading	4.052	4.221	0.065	0.071	−1.095	−75.92	−5.151	−5.838	−1.791	−1.632	−9.405	−9.405
Result	39.69	39.82	0.032	0.033	ND	ND	ND	ND	ND	ND	ND	ND
Mean	39.75		0.033		ND		ND		ND		ND	

ND = Not detected, Results for Ca, Mg are in % and Hg, As, Cd, Pb are in ppm

**Table 4:** Parameters and specifications for manufacturing process of incinerated conch (*Shankha Bhasma*).

Parameters	Specification	Units
Batch quantity	8	Kg
Quantity of fresh lemon juice (of 400 lemons)	7	L
Quantity of diluted lemon juice	32.5	L
pH of fresh juice	2–2.5	-
pH of diluted juice	3–3.5	-
Duration for purification	3	Hr.
Total quantity of aloe vera juice (of 200 mature leaves)	7	Kg
Quantity of aloe vera juice for incineration 1	1.8–2	Kg
Quantity of aloe vera juice for incineration 2	5	Kg
Viscosity of aloe vera juice	2–4	mP
Cow-dung cakes for incineration 1	28	Kg
Cow-dung cakes for incineration 2	22.5	Kg
Total Duration for incineration 1	22	Hr.
Total Duration for incineration 2	19	Hr.
Maximum External Temperature during incineration 1	900–1000	°C
Maximum External Temperature during incineration 2	870–935	°C
Yield of Raw material to Finished Product	90–94	%

**Table 5:** Parameters and specifications of incinerated conch (*Shankha Bhasma*).

Parameters	Specification
Organoleptic characters	White or greyish white colour, no smell and should not taste pungent
Fineness	Fine like Kohl, enters the crevices of finger tips when rubbed between them.
Smoke test	No smoke when burnt on open flame
Loss on drying	Not More Than 0.5% *w*/*w*
Calcium as CaCO_3_ (Titration)	38–40% *w*/*w*
Ca on ICPOES	39–41% *w*/*w*
Mg on ICPOES	0.03–0.04% *w*/*w*
pH of 1% solution in Distilled Water	9–10
Bulk density	0.7000–0.8500 g/cc
Tapped density	1.100–1.500 g/cc
XRD analysis	100% Calcite form
TGA analysis	42–44% loss after 750 °C
FTIR analysis	CO_3_^2−^ peaks at 1410, 874, 712 cm^−1^
SEM analysis	Polygonal particles or agglomerates
Particle size	Effective diameter: 400–750 nm Poly-dispersity: 0.01–0.06 10% <1200 nm (1.2 μm), 50% <2600 nm (2.6 μm), 90% <5500 nm (5.5 μm)
Heavy metals on ICPOES	Hg, As, Cd and Pb- Within permissible limits

**Table 6:** Details of the purification process of conches.

Parameters	Unit	Batch 1	Batch 2	Batch 3
Raw conch	Kg	8	8	8
Time required for washing	H	1.5	1.5	1.5
Fresh lemon juice	L	7.22	7.22	7.22
Water added for dilution	L	25.27	25.27	25.27
Lemon juice diluted	L	32.5	32.5	32.5
Quantity required for boiling	L	32	32	32
Lemon juice residue after boiling	L	26.5	27.3	27.9
Temperature maintained	°C	98–100	99–100	98–100
Weight after purification	Kg	7.950	7.960	7.940

**Table 7:** Quantitative details of the incineration process of conches.

Parameters	Unit	Batch 1	Batch 2	Batch 3
Pots used for each batch	No	04	04	04
Batch quantity	Kg	7.950	7.960	7.940
Purified conch in each pot	Kg	02	02	02
Aloe vera juice in each pot for 1st incineration	Kg	02	02	02
Total Aloe vera juice used for 1st incineration	Kg	08	08	08
Aloe vera juice used for trituration for 2nd incineration	Kg	5	5	5
Triturated conch in each pot for 2nd incineration	Kg	1.875	1.910	1.900
Cow-dung cakes used for 1st incineration	Kg	28	28	28
Cow-dung cakes used for 2nd incineration	Kg	22.5	22.5	22.5

## Supplementary Materials

The following are available online at http://www.mdpi.com/1660-3397/16/11/450/s1, Table S1: Temperature recorded during 1st incineration of conch in all the three batches; Table S2: Temperature recorded during 2nd incineration of conch in all the three batches.

## Appendix A

**Table A1:** Yield of incinerated conch through various stages.

Stage	Time	Batch 1		Batch 2		Batch 3	
		Weight (Kg)	Weight for Next Step (Kg)	Weight (Kg)	Wt. for Next Step (Kg)	Weight (Kg)	Weight for Next Step (Kg)
**Grinding 1**	Before	7.925		7.935		7.920	
	After	7.920	7.910	7.910	7.900	7.900	7.890
**Grinding 2**	Before	7.490		7.640		7.590	
	After	7.485	7.475	7.620	7.610	7.570	7.560
**Trituration 1**	Before	7.910		7.900		7.890	
	After	7.930	7.920	7.920	7.910	7.920	7.910
**Trituration 2**	Before	7.475		7.610		7.560	
	After	7.490	7.480	7.640	7.630	7.595	7.585
**Incineration 1**	Before	7.930		7.910		7.910	
	After	7.515	7.490	7.650	7.640	7.580	7.570
**Incineration 2**	Before	7.480		7.630		7.585	
	After	7.250	7.200	7.450	7.400	7.360	7.320

**Table A2:** Stage-wise loss of moisture, organic matrix and carbon dioxide in TGA.

Sr. No.	Material	Moisture % (100 °C)	Organic Matrix % (450 °C)	Carbon Dioxide % (upto 850 °C)
1	Raw conch	1.179	0.1466	42.71
2	Incinerated conch-Batch 1	0.4482	0.03564	43.20
3	Incinerated conch-Batch 2	0.3040	0.02763	43.28
4	Incinerated conch-Batch 3	0.1647	0.02542	43.23
