# Supplementary material for: Pharmaceutical Standardization and Physicochemical Characterization of Traditional Ayurvedic Marine Drug: Incinerated Conch Shell (Shankha Bhasma)
Source: Mar Drugs. 2018 Nov 15;16(11):450. doi: 10.3390/md16110450 (PMC6266202; doi:10.3390/md16110450)
Supplement: Supplementary file 1 [file marinedrugs-16-00450-s001.pdf]

Table S1: Temperature recorded during 1st incineration of conch in all the three batches (Yellow highlight shows every 1 h temperature used for plotting graph shown in image and red bold highlight show temperature defining the phases of entire incineration process)

Incineration 1					
**Date: Time**	**Batch 1**	**Date: Time**	**Batch 2**	**Date: Time**	**Batch 3**
27-5-16 09:47	36	28-5-16 15:12	33	30-5-16 15:18	35
27-5-16 09:57	36	28-5-16 15:22	33	30-5-16 15:28	35
27-5-16 10:07	38	28-5-16 15:32	34	30-5-16 15:38	37
27-5-16 10:17	38	28-5-16 15:42	34	30-5-16 15:48	37
27-5-16 10:27	40	28-5-16 15:52	35	30-5-16 15:58	37
27-5-16 10:37	40	28-5-16 16:02	35	30-5-16 16:08	39
27-5-16 10:47	42	28-5-16 16:12	36	30-5-16 16:18	39
27-5-16 10:57	65	28-5-16 16:22	35	30-5-16 16:28	40
27-5-16 11:07	91	28-5-16 16:32	38	30-5-16 16:38	41
27-5-16 11:17	136	28-5-16 16:42	38	30-5-16 16:48	41
27-5-16 11:27	167	28-5-16 16:52	40	30-5-16 16:58	44
27-5-16 11:37	213	28-5-16 17:02	40	30-5-16 17:08	44
27-5-16 11:47	267	28-5-16 17:12	42	30-5-16 17:18	46
27-5-16 11:57	294	28-5-16 17:22	69	30-5-16 17:28	74
27-5-16 12:07	313	28-5-16 17:32	92	30-5-16 17:38	87
27-5-16 12:17	358	28-5-16 17:42	139	30-5-16 17:48	118
27-5-16 12:27	401	28-5-16 17:52	178	30-5-16 17:58	167
27-5-16 12:37	476	28-5-16 18:02	259	30-5-16 18:08	242
27-5-16 12:47	521	28-5-16 18:12	313	30-5-16 18:18	365
27-5-16 12:57	596	28-5-16 18:22	424	30-5-16 18:28	383
27-5-16 13:07	659	28-5-16 18:32	536	30-5-16 18:38	401
27-5-16 13:17	710	28-5-16 18:42	601	30-5-16 18:48	542
27-5-16 13:27	**782**	28-5-16 18:52	693	30-5-16 18:58	577
27-5-16 13:37	857	28-5-16 19:02	746	30-5-16 19:08	636
27-5-16 13:47	959	28-5-16 19:12	792	30-5-16 19:18	**813**
27-5-16 13:57	951	28-5-16 19:22	**818**	30-5-16 19:28	836
27-5-16 14:07	912	28-5-16 19:32	875	30-5-16 19:38	873
27-5-16 14:17	876	28-5-16 19:42	916	30-5-16 19:48	890
27-5-16 14:27	834	28-5-16 19:52	933	30-5-16 19:58	901
27-5-16 14:37	**793**	28-5-16 20:02	968	30-5-16 20:08	910
27-5-16 14:47	753	28-5-16 20:12	978	30-5-16 20:18	922
27-5-16 14:57	697	28-5-16 20:22	956	30-5-16 20:28	920
27-5-16 15:07	623	28-5-16 20:32	912	30-5-16 20:38	918
27-5-16 15:17	567	28-5-16 20:42	**856**	30-5-16 20:48	913
27-5-16 15:27	496	28-5-16 20:52	798	30-5-16 20:58	865
27-5-16 15:37	403	28-5-16 21:02	736	30-5-16 21:08	**788**
27-5-16 15:47	340	28-5-16 21:12	677	30-5-16 21:18	701
27-5-16 15:57	312	28-5-16 21:22	629	30-5-16 21:28	682
27-5-16 16:07	276	28-5-16 21:32	583	30-5-16 21:38	613
27-5-16 16:17	243	28-5-16 21:42	514	30-5-16 21:48	527
27-5-16 16:27	197	28-5-16 21:52	459	30-5-16 21:58	464
27-5-16 16:37	173	28-5-16 22:02	413	30-5-16 22:08	381
27-5-16 16:47	150	28-5-16 22:12	374	30-5-16 22:18	356
27-5-16 16:57	141	28-5-16 22:22	326	30-5-16 22:28	335
27-5-16 17:07	129	28-5-16 22:32	293	30-5-16 22:38	282
27-5-16 17:17	113	28-5-16 22:42	245	30-5-16 22:48	267

27-5-16 17:27	109	28-5-16 22:52	228	30-5-16 22:58	234
27-5-16 17:37	101	28-5-16 23:02	217	30-5-16 23:08	215
27-5-16 17:47	97	28-5-16 23:12	205	30-5-16 23:18	197
27-5-16 17:57	95	28-5-16 23:22	192	30-5-16 23:28	181
27-5-16 18:07	92	28-5-16 23:32	180	30-5-16 23:38	178
27-5-16 18:17	88	28-5-16 23:42	174	30-5-16 23:48	168
27-5-16 18:27	85	28-5-16 23:52	164	30-5-16 23:58	161
27-5-16 18:37	83	29-5-16 00:02	159	31-5-16 00:08	152
27-5-16 18:47	81	29-5-16 00:12	152	31-5-16 00:18	147
27-5-16 18:57	81	29-5-16 00:22	145	31-5-16 00:28	141
27-5-16 19:07	80	29-5-16 00:32	144	31-5-16 00:38	134
27-5-16 19:17	80	29-5-16 00:42	144	31-5-16 00:48	133
27-5-16 19:27	79	29-5-16 00:52	141	31-5-16 00:58	126
27-5-16 19:37	78	29-5-16 01:02	141	31-5-16 01:08	122
27-5-16 19:47	78	29-5-16 01:12	139	31-5-16 01:18	118
27-5-16 19:57	75	29-5-16 01:22	138	31-5-16 01:28	117
27-5-16 20:07	74	29-5-16 01:32	138	31-5-16 01:38	116
27-5-16 20:17	74	29-5-16 01:42	132	31-5-16 01:48	112
27-5-16 20:27	71	29-5-16 01:52	129	31-5-16 01:58	108
27-5-16 20:37	71	29-5-16 02:02	124	31-5-16 02:08	104
27-5-16 20:47	69	29-5-16 02:12	120	31-5-16 02:18	102
27-5-16 20:57	69	29-5-16 02:22	119	31-5-16 02:28	100
27-5-16 21:07	68	29-5-16 02:32	115	31-5-16 02:38	99
27-5-16 21:17	67	29-5-16 02:42	112	31-5-16 02:48	99
27-5-16 21:27	67	29-5-16 02:52	110	31-5-16 02:58	97
27-5-16 21:37	67	29-5-16 03:02	107	31-5-16 03:08	97

27-5-16 21:47	66	29-5-16 03:12	105	31-5-16 03:18	96
27-5-16 21:57	65	29-5-16 03:22	104	31-5-16 03:28	95
27-5-16 22:07	65	29-5-16 03:32	101	31-5-16 03:38	92
27-5-16 22:17	64	29-5-16 03:42	98	31-5-16 03:48	88
27-5-16 22:27	64	29-5-16 03:52	96	31-5-16 03:58	85
27-5-16 22:37	63	29-5-16 04:02	95	31-5-16 04:08	83
27-5-16 22:47	63	29-5-16 04:12	91	31-5-16 04:18	82
27-5-16 22:57	63	29-5-16 04:22	91	31-5-16 04:28	74
27-5-16 23:07	62	29-5-16 04:32	88	31-5-16 04:38	73
27-5-16 23:17	62	29-5-16 04:42	88	31-5-16 04:48	73
27-5-16 23:27	61	29-5-16 04:52	88	31-5-16 04:58	71
27-5-16 23:37	61	29-5-16 05:02	86	31-5-16 05:08	69
27-5-16 23:47	60	29-5-16 05:12	86	31-5-16 05:18	69
27-5-16 23:57	60	29-5-16 05:22	86	31-5-16 05:28	67
28-5-16 00:07	60	29-5-16 05:32	81	31-5-16 05:38	65
28-5-16 00:17	59	29-5-16 05:42	81	31-5-16 05:48	65
28-5-16 00:27	59	29-5-16 05:52	77	31-5-16 05:58	62
28-5-16 00:37	59	29-5-16 06:02	77	31-5-16 06:08	62
28-5-16 00:47	58	29-5-16 06:12	75	31-5-16 06:18	56
28-5-16 00:57	58	29-5-16 06:22	75	31-5-16 06:28	56
28-5-16 01:07	57	29-5-16 06:32	73	31-5-16 06:38	56
28-5-16 01:17	56	29-5-16 06:42	73	31-5-16 06:48	55
28-5-16 01:27	56	29-5-16 06:52	68	31-5-16 06:58	55
28-5-16 01:37	55	29-5-16 07:02	68	31-5-16 07:08	54
28-5-16 01:47	55	29-5-16 07:12	64	31-5-16 07:18	54
28-5-16 01:57	55	29-5-16 07:22	64	31-5-16 07:28	54

28-5-16 02:07	55	29-5-16 07:32	63	31-5-16 07:38	53
28-5-16 02:17	54	29-5-16 07:42	63	31-5-16 07:48	53
28-5-16 02:27	54	29-5-16 07:52	63	31-5-16 07:58	53
28-5-16 02:37	54	29-5-16 08:02	61	31-5-16 08:08	51
28-5-16 02:47	54	29-5-16 08:12	61	31-5-16 08:18	51
28-5-16 02:57	53	29-5-16 08:22	60	31-5-16 08:28	50
28-5-16 03:07	53	29-5-16 08:32	60	31-5-16 08:38	50
28-5-16 03:17	53	29-5-16 08:42	58	31-5-16 08:48	49
28-5-16 03:27	52	29-5-16 08:52	58	31-5-16 08:58	49
28-5-16 03:37	52	29-5-16 09:02	57	31-5-16 09:08	49
28-5-16 03:47	51	29-5-16 09:12	57	31-5-16 09:18	49
28-5-16 03:57	51	29-5-16 09:22	57	31-5-16 09:28	47
28-5-16 04:07	51	29-5-16 09:32	55	31-5-16 09:38	47
28-5-16 04:17	50	29-5-16 09:42	55	31-5-16 09:48	46
28-5-16 04:27	50	29-5-16 09:52	55	31-5-16 09:58	46
28-5-16 04:37	50	29-5-16 10:02	54	31-5-16 10:08	45
28-5-16 04:47	49	29-5-16 10:12	54	31-5-16 10:18	45
28-5-16 04:57	49	29-5-16 10:22	54	31-5-16 10:28	45
28-5-16 05:07	49	29-5-16 10:32	53	31-5-16 10:38	44
28-5-16 05:17	49	29-5-16 10:42	53	31-5-16 10:48	44
28-5-16 05:27	47	29-5-16 10:52	53	31-5-16 10:58	43
28-5-16 05:37	47	29-5-16 11:02	52	31-5-16 11:08	43
28-5-16 05:47	45	29-5-16 11:12	52	31-5-16 11:18	43
28-5-16 05:57	45	29-5-16 11:22	52	31-5-16 11:28	43
28-5-16 06:07	45	29-5-16 11:32	50	31-5-16 11:38	42
28-5-16 06:17	44	29-5-16 11:42	50	31-5-16 11:48	42

28-5-16 06:27	44	29-5-16 11:52	49	31-5-16 11:58	42
28-5-16 06:37	43	29-5-16 12:02	49	31-5-16 12:08	41
28-5-16 06:47	43	29-5-16 12:12	48	31-5-16 12:18	41
28-5-16 06:57	43	29-5-16 12:22	48	31-5-16 12:28	41
28-5-16 07:07	43	29-5-16 12:32	48	31-5-16 12:38	41
28-5-16 07:17	41	29-5-16 12:42	47	31-5-16 12:48	41
28-5-16 07:27	41	29-5-16 12:52	47	31-5-16 12:58	41
28-5-16 07:37	41	29-5-16 13:02	46	31-5-16 13:08	41
28-5-16 07:47	41	29-5-16 13:12	45	31-5-16 13:18	41

Table S2: Temperature recorded during 2nd incineration of conch in all the three batches (Yellow highlight shows every 1 h temperature used for plotting graph shown in image and red bold highlight show temperature defining the phases of entire incineration process)

Incineration 2					
**Date: Time**	**Batch 1**	**Date: Time**	**Batch 2**	**Date: Time**	**Batch 3**
3-6-16 10:54	34	5-6-16 10:37	36	7-6-16 10:44	34
3-6-16 11:04	34	5-6-16 10:47	35	7-6-16 10:54	35
3-6-16 11:14	35	5-6-16 10:57	36	7-6-16 11:04	35
3-6-16 11:24	35	5-6-16 11:07	42	7-6-16 11:14	40
3-6-16 11:34	36	5-6-16 11:17	45	7-6-16 11:24	40
3-6-16 11:44	36	5-6-16 11:27	46	7-6-16 11:34	43
3-6-16 11:54	37	5-6-16 11:37	47	7-6-16 11:44	43
3-6-16 12:04	56	5-6-16 11:47	65	7-6-16 11:54	61
3-6-16 12:14	116	5-6-16 11:57	124	7-6-16 12:04	118
3-6-16 12:24	213	5-6-16 12:07	201	7-6-16 12:14	215
3-6-16 12:34	298	5-6-16 12:17	276	7-6-16 12:24	282
3-6-16 12:44	375	5-6-16 12:27	354	7-6-16 12:34	345
3-6-16 12:54	433	5-6-16 12:37	383	7-6-16 12:44	376
3-6-16 13:04	488	5-6-16 12:47	465	7-6-16 12:54	436
3-6-16 13:14	547	5-6-16 12:57	534	7-6-16 13:04	562
3-6-16 13:24	615	5-6-16 13:07	598	7-6-16 13:14	587
3-6-16 13:34	**756**	5-6-16 13:17	**746**	7-6-16 13:24	**755**
3-6-16 13:44	877	5-6-16 13:27	856	7-6-16 13:34	845
3-6-16 13:54	915	5-6-16 13:37	892	7-6-16 13:44	901
3-6-16 14:04	902	5-6-16 13:47	887	7-6-16 13:54	876
3-6-16 14:14	845	5-6-16 13:57	823	7-6-16 14:04	811
3-6-16 14:24	**788**	5-6-16 14:07	**765**	7-6-16 14:14	**751**
3-6-16 14:34	667	5-6-16 14:17	645	7-6-16 14:24	701
3-6-16 14:44	588	5-6-16 14:27	592	7-6-16 14:34	667
3-6-16 14:54	531	5-6-16 14:37	567	7-6-16 14:44	623
3-6-16 15:04	476	5-6-16 14:47	515	7-6-16 14:54	567
3-6-16 15:14	431	5-6-16 14:57	478	7-6-16 15:04	534
3-6-16 15:24	356	5-6-16 15:07	425	7-6-16 15:14	503
3-6-16 15:34	304	5-6-16 15:17	396	7-6-16 15:24	485
3-6-16 15:44	274	5-6-16 15:27	362	7-6-16 15:34	458
3-6-16 15:54	230	5-6-16 15:37	345	7-6-16 15:44	432
3-6-16 16:04	213	5-6-16 15:47	310	7-6-16 15:54	389
3-6-16 16:14	196	5-6-16 15:57	274	7-6-16 16:04	317
3-6-16 16:24	182	5-6-16 16:07	251	7-6-16 16:14	285
3-6-16 16:34	173	5-6-16 16:17	216	7-6-16 16:24	243
3-6-16 16:44	164	5-6-16 16:27	192	7-6-16 16:34	204
3-6-16 16:54	154	5-6-16 16:37	189	7-6-16 16:44	196
3-6-16 17:04	148	5-6-16 16:47	182	7-6-16 16:54	191
3-6-16 17:14	142	5-6-16 16:57	174	7-6-16 17:04	186
3-6-16 17:24	135	5-6-16 17:07	168	7-6-16 17:14	181
3-6-16 17:34	129	5-6-16 17:17	163	7-6-16 17:24	175
3-6-16 17:44	124	5-6-16 17:27	157	7-6-16 17:34	171
3-6-16 17:54	119	5-6-16 17:37	153	7-6-16 17:44	167
3-6-16 18:04	112	5-6-16 17:47	149	7-6-16 17:54	162
3-6-16 18:14	109	5-6-16 17:57	136	7-6-16 18:04	156
3-6-16 18:24	104	5-6-16 18:07	129	7-6-16 18:14	149

3-6-16 18:34	102	5-6-16 18:17	123	7-6-16 18:24	144
3-6-16 18:44	99	5-6-16 18:27	119	7-6-16 18:34	138
3-6-16 18:54	97	5-6-16 18:37	113	7-6-16 18:44	134
3-6-16 19:04	95	5-6-16 18:47	111	7-6-16 18:54	129
3-6-16 19:14	88	5-6-16 18:57	106	7-6-16 19:04	125
3-6-16 19:24	83	5-6-16 19:07	102	7-6-16 19:14	117
3-6-16 19:34	81	5-6-16 19:17	98	7-6-16 19:24	111
3-6-16 19:44	79	5-6-16 19:27	95	7-6-16 19:34	107
3-6-16 19:54	78	5-6-16 19:37	91	7-6-16 19:44	103
3-6-16 20:04	78	5-6-16 19:47	86	7-6-16 19:54	99
3-6-16 20:14	75	5-6-16 19:57	83	7-6-16 20:04	96
3-6-16 20:24	74	5-6-16 20:07	81	7-6-16 20:14	94
3-6-16 20:34	71	5-6-16 20:17	77	7-6-16 20:24	93
3-6-16 20:44	69	5-6-16 20:27	75	7-6-16 20:34	90
3-6-16 20:54	67	5-6-16 20:37	73	7-6-16 20:44	89
3-6-16 21:04	67	5-6-16 20:47	72	7-6-16 20:54	85
3-6-16 21:14	64	5-6-16 20:57	70	7-6-16 21:04	84
3-6-16 21:24	64	5-6-16 21:07	67	7-6-16 21:14	82
3-6-16 21:34	62	5-6-16 21:17	65	7-6-16 21:24	80
3-6-16 21:44	61	5-6-16 21:27	64	7-6-16 21:34	79
3-6-16 21:54	59	5-6-16 21:37	62	7-6-16 21:44	76
3-6-16 22:04	59	5-6-16 21:47	62	7-6-16 21:54	76
3-6-16 22:14	56	5-6-16 21:57	61	7-6-16 22:04	73
3-6-16 22:24	56	5-6-16 22:07	61	7-6-16 22:14	69
3-6-16 22:34	55	5-6-16 22:17	60	7-6-16 22:24	68
3-6-16 22:44	54	5-6-16 22:27	60	7-6-16 22:34	66

3-6-16 22:54	54	5-6-16 22:37	59	7-6-16 22:44	65
3-6-16 23:04	54	5-6-16 22:47	59	7-6-16 22:54	65
3-6-16 23:14	53	5-6-16 22:57	56	7-6-16 23:04	64
3-6-16 23:24	53	5-6-16 23:07	56	7-6-16 23:14	62
3-6-16 23:34	52	5-6-16 23:17	54	7-6-16 23:24	62
3-6-16 23:44	52	5-6-16 23:27	54	7-6-16 23:34	60
3-6-16 23:54	51	5-6-16 23:37	52	7-6-16 23:44	58
4-6-16 00:04	51	5-6-16 23:47	52	7-6-16 23:54	58
4-6-16 00:14	51	5-6-16 23:57	51	8-6-16 00:04	57
4-6-16 00:24	50	6-6-16 00:07	51	8-6-16 00:14	57
4-6-16 00:34	50	6-6-16 00:17	49	8-6-16 00:24	56
4-6-16 00:44	50	6-6-16 00:27	49	8-6-16 00:34	56
4-6-16 00:54	49	6-6-16 00:37	48	8-6-16 00:44	55
4-6-16 01:04	49	6-6-16 00:47	48	8-6-16 00:54	55
4-6-16 01:14	49	6-6-16 00:57	48	8-6-16 01:04	55
4-6-16 01:24	48	6-6-16 01:07	47	8-6-16 01:14	53
4-6-16 01:34	48	6-6-16 01:17	47	8-6-16 01:24	53
4-6-16 01:44	48	6-6-16 01:27	47	8-6-16 01:34	50
4-6-16 01:54	48	6-6-16 01:37	46	8-6-16 01:44	50
4-6-16 02:04	47	6-6-16 01:47	46	8-6-16 01:54	50
4-6-16 02:14	47	6-6-16 01:57	46	8-6-16 02:04	47
4-6-16 02:24	47	6-6-16 02:07	46	8-6-16 02:14	47
4-6-16 02:34	46	6-6-16 02:17	45	8-6-16 02:24	45
4-6-16 02:44	46	6-6-16 02:27	45	8-6-16 02:34	45
4-6-16 02:54	46	6-6-16 02:37	45	8-6-16 02:44	44
4-6-16 03:04	46	6-6-16 02:47	45	8-6-16 02:54	44

4-6-16 03:14	46	6-6-16 02:57	45	8-6-16 03:04	44
4-6-16 03:24	45	6-6-16 03:07	45	8-6-16 03:14	44
4-6-16 03:34	45	6-6-16 03:17	45	8-6-16 03:24	44
4-6-16 03:44	45	6-6-16 03:27	45	8-6-16 03:34	44
4-6-16 03:54	45	6-6-16 03:37	45	8-6-16 03:44	44
4-6-16 04:04	45	6-6-16 03:47	45	8-6-16 03:54	43
4-6-16 04:14	44	6-6-16 03:57	44	8-6-16 04:04	43
4-6-16 04:24	44	6-6-16 04:07	44	8-6-16 04:14	43
4-6-16 04:34	44	6-6-16 04:17	44	8-6-16 04:24	42
4-6-16 04:44	44	6-6-16 04:27	44	8-6-16 04:34	42
4-6-16 04:54	43	6-6-16 04:37	44	8-6-16 04:44	42
4-6-16 05:04	43	6-6-16 04:47	43	8-6-16 04:54	42
4-6-16 05:14	43	6-6-16 04:57	43	8-6-16 05:04	41
4-6-16 05:24	43	6-6-16 05:07	43	8-6-16 05:14	41
4-6-16 05:34	42	6-6-16 05:17	43	8-6-16 05:24	41
4-6-16 05:44	42	6-6-16 05:27	42	8-6-16 05:34	40
4-6-16 05:54	41	6-6-16 05:37	42	8-6-16 05:44	40
